# Fast Determination of Optimal Transmission Rate for Wireless Blockchain Networks: A Graph Convolutional Neural Network Approach

**DOI:** 10.3390/s23136098

**Published:** 2023-07-02

**Authors:** Yucong Ju, Fei Song, Yutao Jiao, Weiyi Wang, Wenting Dai, Yuhua Xu

**Affiliations:** 1 College of Communications Engineering, Army Engineering University of PLA, Nanjing 210007, China; 2 School of Communications and Information Engineering, Nanjing University of Posts and Telecommunications, Nanjing 210003, China

**Keywords:** wireless network, blockchain, graph neural network, deep learning, distributed system

## Abstract

One of the primary challenges in wireless blockchain networks is to ensure security and high throughput with constrained communication and energy resources. In this paper, with curve fitting on the collected blockchain performance dataset, we explore the impact of the data transmission rate configuration on the wireless blockchain system under different network topologies, and give the blockchain a utility function which balances the throughput, energy efficiency, and stale rate. For efficient blockchain network deployment, we propose a novel Graph Convolutional Neural Network (GCN)-based approach to quickly and accurately determine the optimal data transmission rate. The experimental results demonstrate that the average relative deviation between the blockchain utility obtained by our GCN-based method and the optimal utility is less than 0.21%.

## 1. Introduction

In recent years, wireless blockchain networks have emerged as robust and distributed solutions for various blockchain applications [1]. The consensus mechanism, a core component of blockchain, validates messages exchanged among devices in unreliable distributed environments where the data transmission rate plays a crucial role [2]. In this context, higher transmission rates reduce block propagation delays, ensuring secure and efficient blockchain systems in wireless networks. However, higher transmission rates also consume more energy and produce more stale blocks, conflicting with the energy constraints of wireless nodes [3]. Therefore, it is both urgent and essential to develop a method for quickly determining the optimal transmission rate for different wireless network topologies.

However, accurately and quickly determining the optimal transmission rate poses a significant challenge. The impact of varying bandwidth on the network has been considered in [1,4]. Currently, there are some works combining the different deep learning techniques and blockchain. The authors in [5] propose the Deep Reinforcement Learning (DRL) algorithm to dynamically adjust the consensus algorithm used by the blockchain, the block producer, the rate at which blocks are generated, and the block size. The authors analyzed the opportunities and challenges of applying blockchain in IoT. Some other works also combine other (deep) approaches [6,7]. Current studies primarily focus on analyzing the impact of a few specific bandwidth values on throughput, latency, and cost, without taking into account the potential influence of transmission rates on wireless blockchains [8,9]. These studies have inadequately addressed the impact of data transmission rates on blockchain system throughput and stale rates in wireless networks, where limited node energy resources and high stale rates lead to more adverse effects of communication on the blockchain system, and do not fully consider the dynamic changes in wireless network topologies. We summarize some related works on similar topics in Table 1.

Therefore, the main motivation of this research is to address the gap in existing studies by developing a method for accurately and swiftly determining the optimal transmission rate in wireless blockchain networks. To address the above problems, we investigate the impact of data transmission rates on wireless blockchain networks and consider the topology changes. The main contributions of this study are summarized as follows:

(1) Due to the collected data being discrete, if processed directly this will reduce the accuracy of the optimal value, so we adopt the curve fitting method to analyze and propose a continuous wireless blockchain network utility function for the data transmission rate. Considering the convexity and continuity of the constructed utility function, we can obtain the globally optimal transmission rate, which serves as a ground truth label in the subsequent graph neural network training.
(2) Given the dynamic nature of network topology, traditional numerical analysis methods fall short in rapidly determining the optimal transmission rate during the deployment of a wireless blockchain network. To address this, we propose a Graph Convolutional Network (GCN)-based methodology. This approach facilitates the accurate inference of utility function coefficients specific to a blockchain network topology, thereby enabling the swift determination of the optimal transmission rate.
(3) The experimental results validate the effectiveness of our proposed GCN-based methodology. It can attain a utility value that has an average relative deviation of less than 0.21% from the optimal target. These findings underscore the robustness and precision of our proposed approach.

## 2. System Model and Problem Formulation

A blockchain system facilitates the secure and reliable processing of transaction data within a complex wireless environment. This environment incorporates various types of devices, including mobile phones, Unmanned Aerial Vehicles (UAVs), intelligent robots, and more.

### 2.1. Wireless Blockchain Network

In a wireless communication network [10,11], miner nodes are responsible for two crucial steps: generating blocks and then broadcasting them to other miners. Once a consensus is reached regarding the newly generated blocks, they are appended to their respective local blockchains [5]. However, the dynamic nature of the complex wireless blockchain system, coupled with the limited communication resources of nodes, results in unreliable wireless links and a rise in stale blocks. It is important to note that maintaining a low stale rate during block generation is a critical measure of a blockchain system’s reliability, and it profoundly influences the system’s overall throughput.

The Proof of Work (PoW) blockchain comprises peer nodes [12], some of which function as miners tasked with block generation. The fundamental purpose of the PoW consensus mechanism is to guarantee data consistency and consensus security by introducing competition for computational resources among the distributed nodes. The generated block is disseminated throughout the blockchain network and incorporated into the blockchain of each node.

In this article, we focus on a wireless blockchain system utilizing the Proof of Work (PoW) consensus mechanism. We assume a network comprising *N* nodes, denoted as $S=\left\{Z_{1},Z_{2}\cdots Z_{N}\right\}$. Notably, there are *M* mining pools formed by miners, denoted as $B=\left\{Z_{b1},Z_{b2}\cdots Z_{bM}\right\}$, aiming to generate revenue through mining. These nodes form a specific network topology. Due to difficulty of adjusting blockchain, we assume that the default block size $B_{s}$ and block interval *I* are approximately constant. Each node is assumed to operate with the same transmission rate *r* for simplicity of analysis.

### 2.2. Wireless Blockchain Performance Evaluation

This letter concentrates on two primary factors that influence the performance of wireless blockchain networks: transmission rate and network topology.

- Transmission rate ***r***: The transmission rate signifies the amount of data transferred per unit of time. As blockchain technology hinges on distributed computing, the creation of stale blocks is unavoidable. By adjusting the communication conditions, essentially the transmission rate, we can lessen the probability of stale blocks. With a higher transmission rate, the system can reduce transmission latency, thus boosting throughput. However, wireless networks operate under limited resources, and an excessive transmission rate might lead to unnecessary bandwidth and energy consumption.
- Topology and information propagation: Considering the network topology and information propagation in wireless networks is vital due to the requirements for mutual information verification and data synchronization. Various topologies influence propagation differently. In this study, we focus on the gossip protocol, which randomly selects multiple nodes for periodic broadcasting.

The stale block rate and system throughput are crucial performance indicators for a blockchain system. Their behavior heavily depends on the design of the blockchain data structure and the choice of consensus protocol. These two metrics often exhibit an inverse relationship. For instance, in Bitcoin, a transaction only gains confirmation after the creation of six or more successive blocks, thereby enhancing security at the cost of reduced transaction throughput.

- Stale rate s: In a blockchain, a stale block is a block that has been successfully mined but is not included in the main chain because another block was mined at the same height, and that block was added to the main chain first. Stale blocks occur due to network delays or nodes competing to mine the next block. Stale blocks can cause inconsistencies in the blockchain because they contain transactions that are not verified and added to the main chain. The transactions within a stale block that are not added to the main chain are not considered valid, and the corresponding users’ account balances are not updated. If many stale blocks occur frequently, they can build up, and the blockchain will not be able to process transactions as quickly and efficiently as it should. This can cause the blockchain’s maintainability and performance to suffer, making the system less reliable and trustworthy. Furthermore, the existence of a significant number of stale blocks can be exploited by attackers to carry out malicious activities such as double-spending and opportunistic attacks, making the network less secure. Moreover, the occurrence of stale blocks boosts the appeal of the network to malicious nodes, adds to bandwidth overhead, and consumes valuable wireless communication resources. Therefore, to ensure the blockchain’s reliability and performance, it is essential to minimize the occurrence of stale blocks as much as possible.
- ***TPS***: Transaction throughput is quantified by the number of transactions processed per second. In our model, the transmission rate dictates the speed of transaction confirmation, and the transmission latency $t_{s}$ sets an upper limit on the maximum throughput as follows:
  (1) $$\frac{B_{S}}{{\bar{s}}_{tx}\left(t_{s}+I\right)}$$
  where ${\bar{s}}_{tx}$ represents the average transaction size, which is also approximately constant. For example, Bitcoin’s throughput is typically limited to 7 transactions per second (TPS), while Ethereum manages between 20 and 30 TPS. It is worth noting that communication capabilities often serve as a bottleneck in transaction throughput, particularly given the unreliability of communication in a wireless network setting.

### 2.3. Maximizing Utility for Wireless Blockchain Systems

In our experimental data, transmission rates are presented as discrete values. However, the highest value in discrete form does not necessarily represent the global optimum, thus hindering the accurate determination of the optimum transmission rate. We transformed these discrete data points into continuous values using curve fitting to better understand the impact of transmission rates on wireless blockchain performances, including TPS and stale rate. This procedure allows us to deduce the relationship between transmission rate and blockchain performance more accurately. Distinct topologies are represented by different fitting parameters, and the most suitable parameters are determined through GCN training. In the following, we present the utility functions:

(2) $$F_{T}\left(r\right)=a_{1}r^{b_{1}}+c_{1},$$

(3) $$F_{s}\left(r\right)=a_{2}r^{b_{2}}+c_{2},$$

where *a*, *b*, and *c* in the above equations represent curve-fitting parameters. Equations (2) and (3) demonstrate the function’s universality in modeling the TPS and stale rate s. In different network topologies, we obtain varying sets of parameters. Extensive experimental evaluations have shown that this fitting function can achieve an R-squared value exceeding 90%.

From the perspective of wireless blockchain development, there is a dual desire: to maintain a stable, trustworthy system with high throughput and low staleness, while simultaneously preserving limited wireless resources. Although increased transmission rates boost throughput, they also consume resources. Therefore, transmission rate is considered to carry a cost: the higher the transmission rate, the greater the resource consumption. Consequently, the utility function of a wireless blockchain could be formulated as follows:

(4) $$U\left(r\right)=\alpha F_{T}\left(r\right)-\beta F_{s}\left(r\right)-F\left(r\right),$$

where α and β are constant parameters, $F_{T}\left(r\right)$ and $F_{s}\left(r\right)$ are the fitting functions of TPS and stale rate concerning transmission rate, respectively, and F(r) indicates the energy consumption of the transmission rate, which will be introduced in detail in Section 3. According to the above analyses, the wireless blockchain utility maximization problem can be formulated as follows:

maxU,

(5) s.t.0<r≤C.

The objective function *U* is influenced by both the fitting function and the transmission rate, where *C* indicates the upper bound of the transmission rate, i.e., the bandwidth limit.

## 3. GCN-Based Fast Determination of Data Transmission Rate

**Figure 1:**
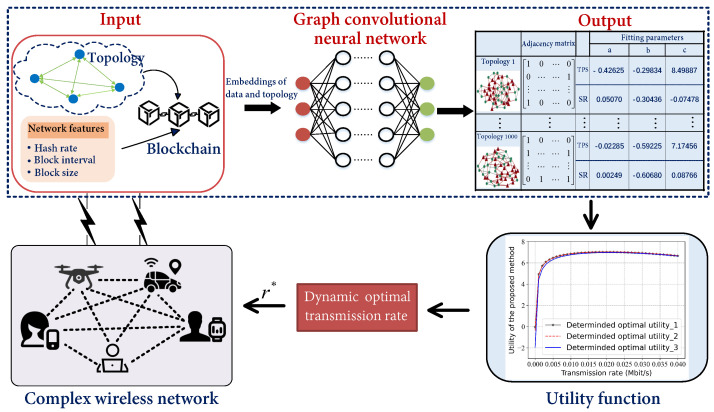
The system framework for the fast determination of GCN-based transmission rate in a wireless blockchain network.

In this section, we introduce a framework predicated on adjusting the transmission rate to address challenges of resource-constrained wireless communication devices and complex, dynamic environments that generate stale blocks, as depicted in Figure 1. With the experimental platform for wireless blockchain systems, we collect a dataset containing the blockchain network running status for various network topologies. Then, we use the dataset to train a graph neural network model which can instantly output the fitting parameters and utility function for the input blockchain network topology, ultimately facilitating a swift and precise determination of the optimal transmission rate.

### 3.1. Graph Convolutional Neural Network Model

Traditional methods often fall short in handling data efficiently and accurately. To tackle these limitations, we introduce a Graph Convolutional Network (GCN)-based approach that effectively mitigates these challenges. This innovative approach allows protocols to adapt more seamlessly to alterations in network topology, thus enhancing resilience towards node failures or network outages. The blockchain network can be conveniently structured into a graph G=(V,E), where *V* is the set of nodes denoted as $V=\left\{v_{1},v_{2}\cdots v_{N}\right\}$, and *E* is the set of edges. Each node v∈V signifies a blockchain node and encapsulates an m-dimensional node feature vector [13]. These node features are represented by the matrix $X\in R^{|V|\times m}$. Each edge e=(u,v)∈E indicates a communication link between the connected nodes.

In our model, the input layer plays a crucial role in preprocessing the dataset. Initially, the topological adjacency matrix *A* of the blockchain network is processed using graph embedding techniques to obtain a vector representation for each node in the graph. The adjacency matrix $A=\left\{a_{ij}=0,1,i,j\in N\right\}$ reflects the communication state between nodes, where $a_{ij}=1$ indicates that nodes *i* and *j* are in a communication state, while $a_{ij}=0$ means no connection between the two nodes. Subsequently, the relevant attribute data of the nodes are organized according to the rules to form the graph structure G=(V,E). The processed graph vectors, along with node features such as hash rate, block interval I, and block size $B_{s}$, are concatenated together and serve as the input to the neural network.

The key function of a multi-layer Graph Convolutional Network (GCN) is to process node features. Our objective is to adjust the parameters in accordance with the network topology through the development of a GCN model [14]. In our model, the updating rule for the representation of the *l*-th layer is

(6) $$H^{l+1}=\sigma \left(AH^{l}W^{l}\right),$$

where $H^{l+1}\in R^{N\times m_{l+1}}$ is the output feature matrix of the layer, *N* is the number of nodes and $m_{l+1}$ is the dimension of the node output feature. $A\in R^{N\times N}$ is the adjacency matrix derived from the input graph, and $H\left(l\right)\in R^{N\times m_{l}}$ represents the feature matrix of the *l*-th layer. *W* is the weight matrix that maps from layer *l* to the (l+1)-th hidden layer, and σ is a non-linear activation function, such as the Rectified Linear Unit (ReLU) function. Graph Convolutional Network (GCN) algorithms allow for the aggregation of feature vectors from neighboring nodes into their node vectors. After passing through multiple convolutional layers, these updated node features are incorporated into the node vectors. These features contain information about nodes with related attributes, enabling us to effectively obtain the fitted parameters.

**Figure 2:**
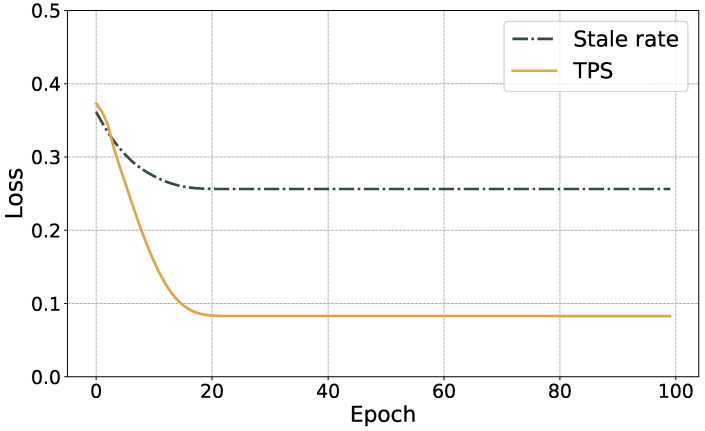
The training loss of the proposed graph neural network.

Furthermore, the performance of the GCN network can be studied for error analysis based on a customized loss function. Unlike the mean squared error (MSE) loss function in the traditional sense, we will randomly select t sample values of the fitting parameters for analysis with the following equation:

(7) $$Loss=\frac{1}{t}\sum_{i=1}^{t}{\left(\hat{y_{i}}-y_{i}\right)}^{2},$$

where ${\hat{y}}_{i}$ is the fitting parameter determined quickly as ${\hat{y}}_{i}\in \left\{\hat{a},\hat{b},\hat{c}\right\}$, $y_{i}$ is the actual fitting parameter value determined as $y_{i}\in \left\{a,b,c\right\}$ and *t* is the number of samples. In Figure 2, we observe that the TPS and stale rate losses converge during training. Our model can generalize to new test data and perform reliably under varying network topologies.

### 3.2. Fast Determination of Transmission Rate

Using the trained Graph Convolutional Network (GCN) parameters, we can rapidly produce fitting curves for any network topology with a network size of *N*. Initial analysis of the experimental data revealed that $a_{1}<0$, $b_{1}<0$, $a_{2}>0$, and $b_{2}<0$. This suggests that the function $F_{T}$ is concave, while $F_{s}$ is a convex function. The difference between these two still yields a concave function. Assuming the transmission energy consumption is a quadratic cost function in relation to effort level, as per [15], we express F(r) as $\lambda r^{2}$, where λ>0. As the second derivative $\frac{\partial^{2}U}{\partial {\left(r\right)}^{2}}$ is negative, our problem is a convex optimization problem. Given Equations (2)–(4), we can express the utility function *U*, which is solely dependent on r, as follows:

(8) $$U\left(r\right)=\alpha \left(a_{1}r^{b_{1}}+c_{1}\right)-\beta \left(a_{2}r^{b_{2}}+c_{2}\right)-\lambda r^{2},$$

where α, β, and λ are constant parameters. The principal problem is to maximize the transmission rate benefit function, and the problem can be formulated as follows:

maxU(r),

(9) s.t.0<r≤C.

From the above analysis, we obtain the optimal transmission rate by using the non-linear least squares method to obtain the optimal transmission rate $r^{*}$.

## 4. Simulation Results

In this section, we investigate the effectiveness of a GCN-based fitting parameter deduced in a Bitcoin trading network and the impact of various parameters on the utility of wireless devices and blockchain networks. We provide simulation and analysis results for the blockchain network at different transmit rates. We describe the experimental setup, including the dataset and experimental metrics, and then present the experimental results in detail. The simulated network setting is configured with the following parameters. There are 50 nodes in the network, and half of those are miners with equal hash rates, so there would be two hash states:

(10) $$h=\left\{\begin{array}{cc} \frac{1}{M}, & if\;node\;is\;miner\\ 0, & else \end{array}\right..$$

The parameters of the blockchain are referenced from Bitcoin [12], which is set to its default value if not otherwise specified. In Bitcoin, the default value of the block size is 1 MB, and the block interval is 10 min. Bitcoin adjusts the difficulty every 2016 blocks (approximately every two weeks) to maintain a target block time, but they can be adjusted to support larger or smaller block sizes based on network requirements. In our system, we assume the average transaction size is 250 B, the block interval is 1 s, the difficulty is adjusted every 10 blocks, and all other parameters are set the same as in Bitcoin.

Considering the generality of the model and the training efficiency, the data are normalized before training. The min–max standardization approach was used to normalize the fitted parameters and map the data to the interval $\left[0,1\right]$. In the experiment, adjusting some parameters will critically affect the training of the network or even the accuracy in the final testing stage. We utilized a combination of manual tuning and an automated search algorithm to determine the optimal values. We initially set our hyperparameters to reasonable values based on prior knowledge and empirical observations in the field. This allows us to establish a baseline performance for our network. Through an iterative process, we carefully adjusted the individual hyperparameters while monitoring the performance of the network. We conducted a number of experiments to systematically explore various combinations of hyperparameter values. To further refine our hyperparameters, we refer to the classical literature to make the results more robust and general [16,17,18].In this letter, the learning rate is set to 0.0001. We use a dataset with 1000 communication network topologies; regarding the division of the dataset, 85% of these were randomly selected for the training set and 15% for the test set. To evaluate the accuracy of the quickly determined utility value, we define the average relative deviation as follows:

(11) $$d=\frac{1}{t}\sum_{i=1}^{t}\frac{\left|u_{f}-u_{o}\right|}{u_{f}},$$

where *d* is the average relative deviation, $u_{f}$ is the fast determined utility value, $u_{o}$ is the optimal utility value, and *t* is the number of samples.

**Figure 3:**
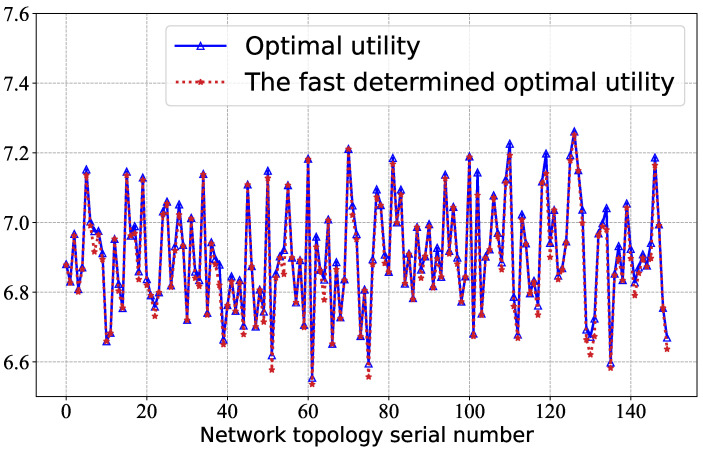
The blockchain utility under different topologies.

Figure 3 compares the fast determined optimal utility values for different topologies with the theoretical optimal utility values. The results determined by the GCN-based approach should be as close as possible to the optimal r. For instance, the theoretical optimal value for the twentieth topology is 6.78, and our determined value of 6.79 is quite close to the theoretical optimal value. For both the determined and theoretical optimal values, we find that the average relative deviation is less than 0.21% according to Equation (11), which indicates that the deviation is small between the values determined by the method and the optimal value.

**Figure 4:**
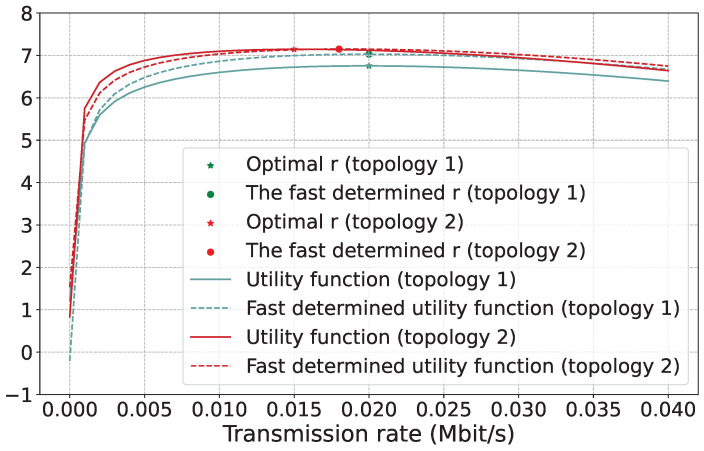
The impact of transmission rate *r*. The optimal *r* means the transmission rate of the maximum utility according to the real fitting data.

We choose topologies and find the relationship between the transmission rate and the utility function value, as shown in Figure 4, with a fit of over 90% between the determined and original utility function. When *r* is less than 0.015, as it increases, more transactions are conducted in the blocks, and the system has higher throughput. At the same time, the blocks mined by miners are instantly propagated to other nodes, causing a lower stale rate, and the cost function value is smaller at this time. When *r* increases to a certain value, higher than around 0.015, the throughput of the system does not consistently increase due to *I* and $B_{S}$ limitations, and the stale rate remains almost stable. However, the value of the cost function keeps increasing as r increases such that there is a peak in the utility function, which inevitably leads to an optimal transmission rate.

## 5. Conclusions

In this study, we presented a GCN-based model to quickly determine node transmission rates in wireless blockchain systems. Specifically, we found that training the fitting parameters can obtain more accurate optimal transmission rates while having a low stale rate and high throughput. Furthermore, we derived the corresponding optimal values from the utility functions based on the obtained parameters; a high degree of accuracy was achieved between the fast determination of the optimal utility values and the theoretical original utility values, demonstrating the reliability and validity of the approach. In summary, our research provided valuable insights into wireless blockchain systems.

In future work, we will explore heterogeneous topologies to build a trusted environment for next-generation computing, and study the effects of different wireless network settings on model performance. The proposed model assumes that the wireless network operates under ideal conditions, such as no interference or collisions. Future research could explore how the model performs in more realistic settings, such as in the presence of interference or varying traffic load, and investigate how to incorporate real-time feedback on network performance into the model. The proposed model is trained on historical data, but in practice, real-time feedback on network performance may be necessary. Future research could explore ways to incorporate this feedback into the model to improve its accuracy and responsiveness.

## Figures and Tables

**Table 1:** Related works on similar topics.

Application Field	Ref.	Year	Mentioned Issue
Internet of Things and blockchain	[1]	2018	The interactions in blockchain would involve an increase in bandwidth.
Adaptive adjustment in blockchain	[4]	2022	Changes in bandwidth affect the stale rate.
Wireless blockchain communication resources	[5]	2021	Larger available bandwidth can lead to a low latency.
Blockchain and security	[6]	2020	System sets the value based on the bandwidth and other parameters.

## Data Availability

Not applicable.
